# Sterile protection and transmission blockade by a multistage anti-malarial vaccine in the pre-clinical study

**DOI:** 10.3389/fimmu.2022.1005476

**Published:** 2022-09-29

**Authors:** Mitsuhiro Iyori, Andrew M. Blagborough, Tetsushi Mizuno, Yu-ichi Abe, Mio Nagaoka, Naoto Hori, Iroha Yamagoshi, Dari F. Da, William F. Gregory, Ammar A. Hasyim, Yutaro Yamamoto, Akihiko Sakamoto, Kunitaka Yoshida, Hiroaki Mizukami, Hisatoshi Shida, Shigeto Yoshida

**Affiliations:** ^1^ Laboratory of Vaccinology and Applied Immunology, Kanazawa University School of Pharmacy, Kanazawa University, Ishikawa, Japan; ^2^ Department of Pathology, University of Cambridge, Cambridge, United Kingdom; ^3^ Department of Parasitology, Graduate School of Medical Sciences, Kanazawa University, Ishikawa, Japan; ^4^ Département de Biologie Médicale et Santé Publique, Unité Paludisme et Maladies Tropicales Négligées, Institut de Recherche en Sciences de la Santé, Bobo-Dioulasso, Burkina Faso; ^5^ Division of Gene Therapy, Jichi Medical University, Tochigi, Japan; ^6^ Institute for Genetic Medicine, Hokkaido University, Hokkaido, Japan

**Keywords:** malaria, vaccine, *plasmodium falciparum*, PfCSP, Pfs25, LC16m8Δ, adeno-associated virus (AAV)

## Abstract

The Malaria Vaccine Technology Roadmap 2013 (World Health Organization) aims to develop safe and effective vaccines by 2030 that will offer at least 75% protective efficacy against clinical malaria and reduce parasite transmission. Here, we demonstrate a highly effective multistage vaccine against both the pre-erythrocytic and sexual stages of *Plasmodium falciparum* that protects and reduces transmission in a murine model. The vaccine is based on a viral-vectored vaccine platform, comprising a highly-attenuated vaccinia virus strain, LC16m8Δ (m8Δ), a genetically stable variant of a licensed and highly effective Japanese smallpox vaccine LC16m8, and an adeno-associated virus (AAV), a viral vector for human gene therapy. The genes encoding *P. falciparum* circumsporozoite protein (PfCSP) and the ookinete protein P25 (Pfs25) are expressed as a Pfs25–PfCSP fusion protein, and the heterologous m8Δ-prime/AAV-boost immunization regimen in mice provided both 100% protection against PfCSP-transgenic *P. berghei* sporozoites and up to 100% transmission blocking efficacy, as determined by a direct membrane feeding assay using parasites from *P. falciparum*-positive, naturally-infected donors from endemic settings. Remarkably, the persistence of vaccine-induced immune responses were over 7 months and additionally provided complete protection against repeated parasite challenge in a murine model. We propose that application of the m8Δ/AAV malaria multistage vaccine platform has the potential to contribute to the landmark goals of the malaria vaccine technology roadmap, to achieve life-long sterile protection and high-level transmission blocking efficacy.

## Introduction

Malaria has had a profound effect on human health for thousands of years and remains one of the most serious, life-threatening infectious diseases. Despite past and ongoing efforts to control and reduce the mortality and morbidity caused by this disease, 241 million people were estimated to be infected in 2020, with deaths estimated at 627,000 (1). To exacerbate this situation further, the COVID-19 pandemic disrupted ongoing malaria services, leading to a marked increase in cases and deaths (2).

The recent (October 2021) endorsement of the RTS,S/AS01 malaria vaccine for broad use in the field is positive news, and is the first anti-malarial vaccine candidate (and the first vaccine to address human parasitic infection) to achieve this key approval milestone. From preliminary data, the inclusion of RTS,S with currently-used chemopreventative treatment (sufadoxine-pyrimethamine plus amodiaquine) results in significantly increased efficacy against clinical malaria, reduced hospital admission rates with severe malaria and reduced mortality rates (3). These findings give a clear indication as to the wide and undeniable benefits of incorporating RTS,S within currently existing anti-malarial control measures. Conversely, it is also widely predicted that the incorporation of RTS,S alone into current control measures will not achieve our previously stated aims against malaria, namely long-term control or elimination, because of limited and waning efficacy (4). As a result, the further development of “second-generation” anti-malarial vaccines, with enhanced characteristics, is key for long-term anti-malarial control or eradication.

To achieve the pressing demand for high, long-lasting malaria vaccine efficacy, we have developed a highly effective and durable next-generation multistage malaria vaccine expressing a fusion protein combining *Plasmodium falciparum* circumsporozoite protein (PfCSP) and Pfs25, that is effective against both pre-erythrocytic stage and sexual-stage parasites based on an LC16m8Δ (m8Δ)/adeno-associated virus (AAV) vaccine platform. M8Δ is a genetically stable variant of a licensed and highly effective Japanese smallpox vaccine LC16m8 (5, 6), and AAV is a viral vector for gene therapy (7). As demonstrated here, the combination of m8Δ and AAV delivery of the fused parasite proteins results in potent inhibition of malarial transmission from vertebrate to mosquito and mosquito to vertebrate, *in vivo*, as examined in the laboratory, and *ex vivo*, as examined by membrane feeding assays performed using parasites from *P. falciparum*-positive, naturally-infected donors from endemic settings. In addition, the simple two-dose immunization regimen described here would potentially be suitable for rapid integration into the current clinical Expanded Programme on Immunization (EPI) vaccines used to immunize infants to achieve high-level efficacy.

## Materials and methods

### Ethics statement

The animal study was reviewed and approved by the Animal Care and Ethical Review Committee of Kanazawa University (No. AP-214212) in Japan. All UK animal works in this study were carried out according to the Animals (Scientific Procedures) Act 1986 Amendment Regulations 2012 (SI 2012/3039) with approval from the University of Cambridge Ethical Review Committee (PPL PP8679814). The Office of Laboratory Animal Welfare Assurance for the University of Cambridge covers all Public Health Service supported activities involving live vertebrates in the US (no. A5634-01). All efforts were made to minimize animal suffering during the experiments. The human study was reviewed and approved by the Centre Muraz Institutional Ethical Committee (Protocol 003-2009/CE-CM), Burkina Faso.

### Vaccine construction and production

To newly construct a mutant that completely lacks the *B5R* gene of LC16mO vaccinia virus (GenBank accession number: AY678277), we performed inverse PCR using pPS : HRΔB5R as a template with primers: 5′-TAACACTGTCGAGCACTAAAAGG-3′ and 5′-TAAATCCGTTAAAATAATTAATAATTA-3′ to produce pBRΔB5R2 in 30 cycles. Then, we transfected pBRΔB5R2 into LC16mO-infected BHK-21 cells (kind gifts from Prof. Samuel Dales, University of Western Ontario, Canada) using a standard method. The progeny viruses were used to infect a monolayer of RK13 cells (kind gifts from Chiba Serum Institute, Japan) and small plaques were selected. After plaque purification, deletion of the *B5R* gene was confirmed by sequencing. The mutant was designated LC16m8Δ2 and was used throughout this study instead of the original mutant LC16m8Δ. The *in vitro* characteristics of LC16m8Δ2 and LC16m8Δ, such as proliferation capacity and plaque size, were indistinguishable.

The transfer vector for m8Δ-Pf(P7.5-CSP)-HA was constructed using a pVR1 plasmid containing a p7.5 promoter (8). The *pfcsp-g* gene (GenBank accession numbers: MB421778 and MB421779) in pENTR-CAG-sPfCSP2-G (9, 10), which encodes PfCSP fused with the transmembrane region of VSV-G protein, included the “TTTTTCT” vaccinia transcription terminator in the open reading frame. To replace the “TTTTTCT” sequence with “TTTCTTC” encoding the same amino acids, pUC57-Simple-VV-VSV-Nhe containing synthetic DNA with the sequence “TTTCTTC” and a *Nhe* I digestion site, was digested with *Pst* I and *Hin*d III and cloned into the *Pst* I and *Hin*d III sites of pENTR-CAG-sPfCSP2-G (pENTR-CAG-sPfCSP2-G2-sWPRE-Nhe). The plasmid pUC57-Simple-VV-WPRE that included digestion sites, such as *Age* I, *Nco* I, *Not* I and *Mfe* I, was prepared by GeneScript (Piscataway, NJ). An *Nco* I-digested fragment of pENTR-CAG-sPfCSP2-G2-sWPRE-Nhe was cloned into the *Nco* I site of pUC57-Simple-VV-WPRE. The resultant plasmid pUC57-Simple-sPfCSP2-VV was digested with *Age* I and *Mfe* I and then cloned into the multi-cloning site with the *Xma* I and *EcoR* I sites of pVR2. The fragment of VSV-G TM that contained digestion sites of *Xma* I and *Fse* I was amplified by PCR with the primers pVSV-G-F1 (5′-CACCCGGGCGTTCGAACATCCTCACATTCAAGAC-3′) and pVSV-G-R7 (5′-TTTTTGGCCGGCCTTACTTTCCAAGTCGGTTCATC-3′) in 30 cycles and cloned into a pCR2.1-TOPO vector (Thermo Fisher Scientific, Waltham, MA). The resultant plasmid pCR-VSV-G-TAA-Fse was digested with *Xma* I and *Fse* I, and then the fragment was cloned into the *Xma* I and *Fse* I sites of pVR1-sPfCSP2-VV. The resulting plasmid, pVR1-sPfCSP2-WPRE(˗)-VV, contained the 7.5 promoter, genes encoding the signal sequence, a FLAG epitope tag and *pfcsp-g*, but not the *wpre* sequence (11), corresponding to the forward direction of the flanking promoter of the vaccinia virus hemagglutinin (HA) gene (**Supplementary Figure S1**) (8). To construct the transfer vector for m8Δ-Pf(P7.5-s25-CSP)-HA, the gene segment encoding PfCSP was replaced with that encoding the Pfs25–PfCSP fusion protein (12). To generate recombinant vaccinia viruses, BHK-21 cells were infected with canarypox virus, and then transfected with the transfer vector and purified genomic DNA of LC16m8Δ2. RK13 cells were exposed to the lysates of transfectants to develop plaques, and then incubated with the peripheral blood of a white leghorn chicken. Recombinant viruses from HA-negative plaques were further amplified and purified for 10 cycles, and the resultant virus exhibited >99.99% purity. The production of viral vectors based on AAVs and Ad is described elsewhere (9, 12, 13).

### Viral transduction

Viral transduction studies were conducted as described previously (12–14). Briefly, HEK293T cells were transduced with m8Δ at a multiplicity of infection (MOI) of 5 or with AAV at a MOI of 10^5^ for 24 h. For western blotting, cell lysates were loaded onto 10% sodium dodecyl sulfate polyacrylamide gels. Proteins were blotted onto an Immobilon FL^®^ PVDF membrane (Merck Millipore, Darmstadt, Germany), and then blocked using 5% skim milk in PBS. Anti-PfCSP monoclonal antibody (mAb) 2A10 or anti-Pfs25 mAb (4B7) was used as the primary antibody, followed by IRDye 800-conjugated goat anti-mouse IgG secondary antibody (Rockland Immunochemicals, Gilbertsville, PA) for detection. The membrane was visualized using an Odyssey infrared imager (LI-COR). For indirect immunofluorescence, an eight-well chamber slide was used for the cell culture. After transduction, the cells were fixed with either 100% methanol for intracellular staining, or 4% paraformaldehyde for cell-surface staining. The fixed cells were blocked with 10% normal goat serum in PBS, and stained with Alexa Fluor 488-conjugated anti-PfCSP mAb (2A10) and Alexa Fluor 594-conjugated anti-Pfs25 mAb (4B7). The slide was mounted with a drop of VECTASHIELD containing 4′,6-diamidino-2-phenylindole (DAPI; Vector Laboratories, Burlingame, CA). The images were acquired using an LSM710 inverted laser scanning microscope (Carl Zeiss, Gottingen, Germany) with a 20× objective. For flow cytometry to analyze cell-surface expression, transduced cells were incubated with 1% FBS/PBS, and then stained with fluorescein-conjugated anti-PfCSP mAb (2A10) and allophycocyanin (APC)-conjugated anti-Pfs25 mAb (4B7). The cells were fixed with 4% paraformaldehyde, washed, and then the fluorescence of the cells was examined by a FACSVerse™ flow cytometer (BD Biosciences, San Jose, CA) and analyzed with FlowJo™ software (version 10, BD Biosciences).

### Immunization

Seven-week-old female Balb/c mice and ICR mice were purchased from Japan SLC (Japan) or Harlan (UK). For virus infection, 1 × 10^7^ PFU of recombinant m8Δ viruses were inoculated by tail scarification (15), and 1 × 10^10^ viral genomes of recombinant AAVs were intramuscularly administered 42 days after the priming immunization. Intramuscular immunization by Ad (5 × 10^7^ PFU) was conducted as previously described (9). Seven to ten mice were used in the challenge infection assays, and three to five mice were used in the transmission blocking assays.

### Elisa

The levels of total mouse IgG specific to PfCSP and Pfs25 were quantified by ELISA as described previously (12). Briefly, ELISA plates were coated with 0.4 µg per well of rCSP or 0.2 µg per well of recombinant Pfs25 overnight, and then blocked with 1% BSA in PBS for 1 h. Tail vein blood samples in 1% BSA in PBS were incubated for 1 h at room temperature, and then incubated for 1 h with horseradish peroxidase (HRP)-conjugated goat anti-mouse IgG (H+L) antibody (Bio-Rad). For determination of the IgG subclasses, HRP-conjugated rabbit anti-mouse IgG1, IgG2a and IgG2b antibodies (Zymed Laboratories, South San Francisco, CA) were used. The plates were developed with a peroxidase substrate solution [H_2_O_2_ and 2,2′-azino-bis(3-ethylbenzothiazoline-6-sulfonate)]. The endpoint titers were expressed as the reciprocal of the highest dilution for which the optical density at 414 nm was equal to 0.15 U, which was above the value of the negative controls (<0.1). All mice used in our experiments were seronegative before immunization.

### ELISpot and flow cytometry

The PfCSP-specific cellular immune response was measured by an ELISpot assay. Ammonium chloride potassium lysing buffer-treated splenocytes were cultured for 20–24 h on BD ELISPOT plates with 1 µg/ml of the immunodominant H-2K^d^-restricted T-cell epitope (NYDNAGTNL, PfCSP_39–47_). The IFN-γ ELISPOT assay was conducted with coating and detecting mAbs from the BDTM IFN-γ ELISPOT Set (BD Biosciences), following the manufacturer’s protocol. Spots were counted with an ELISPOT plate counter (Autoimmun Diagnostika, Strassberg, Germany) and expressed as IFN-γ spot-forming units (SFU) per million splenocytes, after the background subtraction of wells containing cells and medium, but no peptide.

Intracellular cytokine staining for multiple cytokines was conducted by flow cytometry. The splenocytes were stimulated with a final concentration of 1 µg/ml of the H-2K^d^-restricted PfCSP_39–47_ epitope and 1 μg/ml of GolgiPlug™ (BD Biosciences) in a 96-well U-bottom tissue culture plate (Corning Inc., Corning, NY)) for 6 h. The surface markers were antibody-stained in the presence of TruStain FcX™ antibody and normal mouse IgG (Jackson Immuno Research, West Grove, PA), and then the intracellular cytokines were stained in perm buffer (BioLegend) after fixation by 4% paraformaldehyde (Sigma). The following mAbs were used in the designated combinations: anti-CD3ε antibody (clone 145-2C11; PE/Cy7), anti-CD4 antibody (clone GK1.5; APC/Fire™ 750), anti-CD8b antibody (clone YTS156.7.7; PerCP-Cy5.5), anti-IFN-γ antibody (clone XMG1.2; APC), anti-TNF-α antibody (clone MP6-XT22; Brilliant Violet 421™) and anti-IL-2 antibody (clone JES6-5H4; Brilliant Violet 510™). All antibodies were purchased from BioLegend (San Diego, CA).

The number of liver-resident memory CD8^+^ T cells was determined 28 days after the last immunization. The livers in living mice were perfused with buffer 2 (66.74 mM NaCl, 6.71 mM KCl, 6.31 mM CaCl_2_, 100 mM HEPES, 0.226 mM BSA) containing collagenase type IV (0.53 mg/ml, Sigma-Aldrich, St. Louis, MO), and single-cell suspensions were harvested by homogenization using frosted glass. The cells were passed through a 100-μm mesh, resuspended in 35% Percoll/PBS, and centrifuged at 500 ´*g* for 20 min at room temperature. The red blood cells were subsequently lysed. Spleen cells were filtered through a 40-μm mesh and the buffy coat layer resulting from density gradient centrifugation with 15% OptiPrep (Abbott Diagnostics Technologies AS, OSLO, Norway) in PBS was collected. After the number of cells in the pellets and supernatants, excluding debris, was counted, the cells were antibody-stained in the presence of TruStain FcX™ antibody and normal mouse IgG. The following mAbs were used in the designated combinations: anti-CD45 antibody (clone 30-F11; FITC), anti-CD8b antibody (clone YTS156.7.7; PerCP-Cy5.5), anti-CD44 antibody (clone IM7; Brilliant Violet 510™), anti-CD62L antibody (clone MEL-14; APC), anti-CD69 antibody (clone H1.2F3; Brilliant Violet 421™), KLRG1 (MAFA, clone 2F1/KLRG1; APC/Cy7) and the H-2K^d^-restricted PfCSP_39–47_ epitope-bound tetramer (provided by the National Institutes of Health Tetramer Core, Atlanta, GA; PE). All antibodies were purchased from BioLegend.

All data were acquired with a FACSVerse™ flow cytometer (BD Biosciences) and analyzed with FlowJo™ software. The number of leukocytes per gram of tissue was calculated based on the percentage of CD45^+^ cells. The gating strategy was as described previously (14).

### Challenge infection

Challenge was performed by two independent routes, intravenous injection of sporozoites or mosquito bite. Intravenous challenge infection was performed by administration of 1,000 or 2,500 PfCSP/Pb (16) sporozoites either 4–6 weeks after the last immunization for short-term studies or after more than 14 weeks for long-term studies. Challenge infection *via* mosquito bite was conducted by exposure of mice at day 21 post-infection to starved PfCSP/Pb-infected mosquitoes to enable blood feeding. Each mouse was initially exposed to seven mosquitoes for 20 min, following which, all mosquitoes were dissected to assess the presence of sporozoites in the salivary glands, with exposure and dissection repeated until the mouse received sufficient numbers (3 to 7) of confirmed infected bites. Multiple challenge infection was conducted by intravenous administration of 2,500 or 10,000 PfCSP/Pb sporozoites. Tail blood smearing was performed daily from 4 days post-infection to evaluate the development of parasitaemia up to day 14. The prepatent period was defined as the time to reach 1% parasitaemia (9).

### Transmission blocking assay

Direct feeding assays for TB efficacy in vaccinated mice were conducted by inoculating 1 ´ 10^6^ *Pb-*Pfs25DR3 (17)-parasitized red blood cells intraperitoneally into phenylhydrazine-treated mice at 3 days before mosquito feeding. At 3 days post-infection, approximately 60 starved *Anopheles stephensi* mosquitoes were allowed to feed on each infected mouse. On day 10–12 post-feeding, the midguts from the blood-fed mosquitoes were dissected, and the oocyst-positive percentage and intensity were recorded. Percent (%) inhibition of the mean oocyst intensity (transmission-reducing activity; TRA) was calculated as follows: 100 ´ [1 ˗(mean number of oocysts in the test group/mean number of oocysts in the control group)]. The % inhibition of the oocyst-positive percentage (TB activity; TBA) was evaluated as: 100 ´ [1 ˗(proportion of mosquitoes with any oocysts in the test group)/(proportion of mosquitoes with any oocysts in the control group)].

Field-relevant TB efficacy was determined by direct membrane feeding assays on naturally-infected volunteers as previously described (18). Briefly, children aged between 5 and 11 years in Bobo-Dioulasso, Burkina Faso, were screened for the presence of sexual-stage *P. falciparum* parasites by thick blood smears. Informed consent from parents or guardians was obtained for children positive for gametocytes (Protocol 003-2009/CE-CM, Centre Muraz institutional ethical committee). Then, 10 ml of blood was drawn into heparinized tubes to obtain gametocytes (n = 3). Plasma from the gametocyte-positive donor blood was mixed with the relevant mouse sera (harvested at day 115 post-immunization, n = 3) or control sera, at the desired concentration, and was fed to field-caught starved female *An. coluzzii via* a parafilm^®^ membrane. The final immune serum concentrations examined were 1:5, 1:10 and 1:100. Sera from a group of mice vaccinated with the (isotypic) parent m8Δ and AAV1-Luc (13) vectors were used as negative controls (Pooled sera from 3 control mice). Infected mosquitoes were then maintained at 26°C and 80% relative humidity. After 7 days, the midguts from approximately 10–20 mosquitoes were dissected, oocysts were counted and the number of infected mosquitoes was recorded. TRA and TBA were calculated as described above.

### Statistical analysis

To assess the sterile protective efficacy of the PEV vaccines, the numbers of protected and infected mice in the vaccinated group were compared with those in the PBS or naïve control groups by Fisher’s exact tests. A statistical significance test for survival analysis at 1% parasitaemia was performed using the log-rank (Mantel–Cox) test. For the comparison of multiple group differences to antibody responses and cellular immune responses, Kruskal–Wallis tests with Dunn’s correction or repeated measures two-way ANOVA with Sidak’s multiple comparisons test for multiple comparisons were conducted, while Mann–Whitney tests were used to determine differences between two groups. TRA was analyzed by the Mann–Whitney test, and TBA was analyzed by Fisher’s exact test. All statistical analyses were performed using GraphPad Prism software (version 9, San Diego, CA) in which *p* < 0.05 was considered statistically significant. In all figures, *p* values are shown as follows: *, *p* < 0.05; **, *p* < 0.01; ***, *p* < 0.001; ****, *p* < 0.0001.

## Results

### Vaccine construction and optimization

The vaccinia virus LC16m8Δ expressing PfCSP [m8Δ-Pf(P7.5-CSP)-HA] was generated *de novo*. The gene cassette encoding full-length *pfcsp* was fused with the transmembrane region of the G protein of vesicular stomatitis virus (G-TM) to allow PfCSP to be displayed on the cell surface efficiently and integrated within the *hemagglutinin* gene in m8Δ (**Supplementary Figure S1**). Successful recombinant clones were assessed by hemagglutinin tests, plaque ELISA and western blotting (**Supplementary Figures S2A–C**). An indirect immunofluorescence assay showed that PfCSP was expressed both in the cytoplasm of the transduced cells and on the cell surface (**Supplementary Figure S2D**). The m8Δ-Pf(P7.5-CSP)-HA was used to immunize mice in combination with AAV-PfCSP, an AAV type I that expressed the transmembrane form of PfCSP, or AAV(G˗)-PfCSP, which lacked G-TM (13) (**Supplementary Figure S1**), and the protective efficacy was assessed by challenge with PfCSP/*Plasmodium berghei* (Pb) transgenic parasites that expressed PfCSP instead of PbCSP (16) (500 sporozoites, intravenous challenge).

The m8Δ-Pf(P7.5-CSP)-HA prime and AAV(G˗)-PfCSP boost method [m8Δ/AAV(G˗)-PfCSP] provided relatively high protective efficacy (40%) (**Supplementary Figure S3A**). The splenocytes from protected mice induced interferon (IFN)-γ and tumor necrosis factor (TNF)-α secretion specific to H-2K^d^-restricted PfCSP epitope peptide (**Supplementary Figures S3B, C**). M8Δ/AAV-PfCSP provided higher protection than m8Δ/AAV(G˗)-PfCSP, with both m8Δ/AAV(G˗)-PfCSP and m8Δ/AAV-PfCSP inducing significantly higher levels of circulating anti-PfCSP IgG than m8Δ/m8Δ (**Supplementary Figures S3D, E**). Of note, m8Δ/AAV-PfCSP induced a high IgG2a titer, a cytophilic antibody subclass known to produce strong protection (9) (**Supplementary Figure S3F**). On the basis of these results, an m8Δ/AAV method was used in subsequent studies.

**Figure 1:**
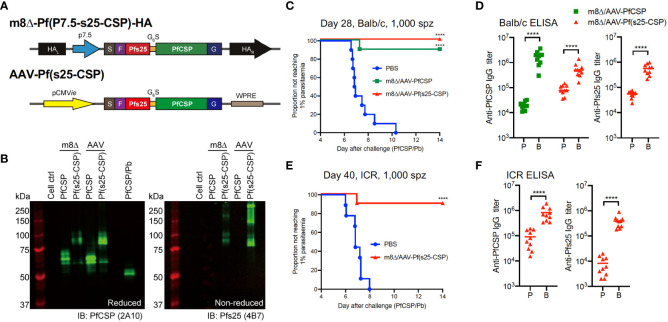
Protective efficacy against low-dose sporozoites. **(A)** Construction of recombinant m8Δ and AAV. HA, hemagglutinin; p7.5, 7.5 promoter; pCMVie, CMV immediate early promoter; S, signal sequence; F, FLAG epitope tag; G6S, GGGGGGS hinge sequence; G, VSV-G TM; WPRE, woodchuck hepatitis virus posttranscriptional regulatory element. **(B)** Western blotting of viral-transduced HEK293T cells. **(C, E)** Protective efficacy in Balb/c mice **(C)** and ICR mice **(E)** immunized with m8Δ/AAV-Pf(s25-CSP) (n = 10) and m8Δ/AAV-PfCSP (n = 10) against 1,000 PfCSP/Pb sporozoites at day 28 (Balb/c) or day 40 (ICR) after the last immunization. *P* values were calculated by log-rank (Mantel–Cox) tests versus the PBS group. **(D, F)** IgG antibodies against PfCSP and Pfs25 at 41 days after priming “P” and 27 days after boosting “B” in Balb/c mice **(D)**, or 41 days after priming and 39 days after boosting in ICR mice **(F)**. *P* values were calculated using Mann–Whitney tests. ****p < 0.0001.

Utilizing this preliminary data, we subsequently developed a multistage malaria vaccine (targeting both PfCSP and the ookinete/macrogamete protein Pfs25) based on the adenovirus (Ad) and AAV delivery platforms, which previously elicited a high level of protection against sporozoite challenge and strong transmission blocking (TB) activity (12). We specifically constructed bivalent vaccines based on m8Δ and AAV that expressed both Pfs25 and PfCSP as a fusion protein with a G_6_S linker [m8Δ-Pf(P7.5-s25-CSP)-HA and AAV-Pf(s25-CSP), respectively] (**Figure 1A**). Western blotting showed that both viral vectors were able to express a Pfs25–PfCSP fusion protein (**Figure 1B**), and cell-surface expression of PfCSP and Pfs25 was confirmed (**Supplementary Figure S4**). Subsequently, experiments were designed to determine both the protective efficacy and TB efficacy of m8Δ-prime/AAV-boost vaccination [m8Δ/AAV-Pf(s25-CSP)] (**Supplementary Figure S5**).

### Protective efficacy

The protective efficacy of vaccines was determined by induction of sterile protection against infection following intravenous challenge with 1,000 transgenic PfCSP/Pb sporozoites. All control Balb/c mice were infected as expected, while 10/10 mice (100%, *p* < 0.0001) were protected in the m8Δ/AAV-Pf(s25-CSP) group and 9/10 mice (90%, *p* = 0.0001) were protected in the m8Δ/AAV-PfCSP group (**Figure 1C**). Both m8Δ/AAV-PfCSP and m8Δ/AAV-Pf(s25-CSP) induced significantly higher anti-PfCSP IgG titers after boosting (19,550 after priming, 1,755,000 after boosting, *p* < 0.0001; 87,200 after priming, 560,500 after boosting, *p* < 0.0001; respectively) (**Figure 1D**). The m8Δ/AAV-Pf(s25-CSP) vaccine also induced a significantly higher anti-Pfs25 IgG titer after boosting (54,693 after priming, 558,165 after boosting, *p* < 0.0001) (**Figure 1D**). CD8^+^ T cells from splenocytes in the m8Δ/AAV-PfCSP-vaccinated mice significantly induced multifunctional T cell markers, such as IFN-γ, IL-2 and TNF-α, in response to the H-2K^d^-restricted PfCSP epitope when compared with those of the naïve and m8Δ/AAV-Pf(s25-CSP) groups (**Supplementary Figure S6A, B**). The numbers of CD8^+^ T cells with memory T cell markers, such as central memory T cells (T_CM_), effector memory T cells (T_EM_) and effector T cells, were significantly increased in the spleens of the m8Δ/AAV-Pf(s25-CSP)-vaccinated mice compared with those in naïve mice (**Supplementary Figure S6C**). The same experiment was also conducted in an outbred strain ICR to limit the effects of MHC restriction and immunodominance in the inbred strain Balb/c. In all, 9/10 (90%, *p* = 0.0001) of the m8Δ/AAV-Pf(s25-CSP)-vaccinated ICR mice were protected, and potent induction of anti-PfCSP and anti-Pfs25 antibodies was also observed in ICR mice (**Figures 1E, F**).

**Figure 2:**
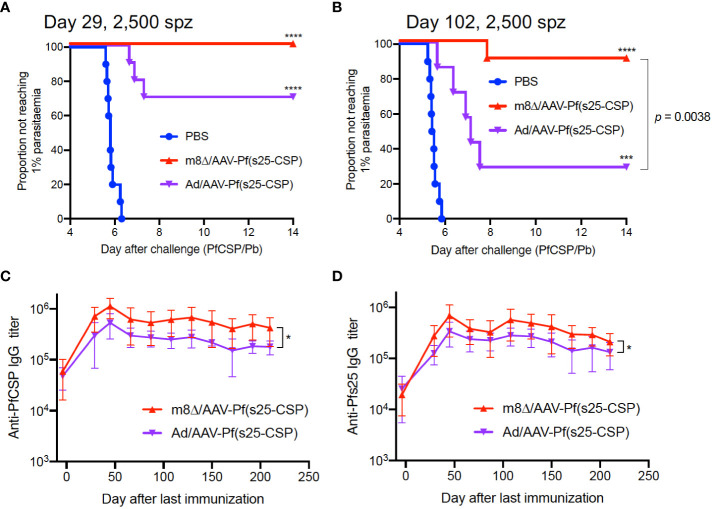
Protective efficacy against high-dose sporozoites. **(A, B)** Protective efficacy in Balb/c mice immunized with m8Δ/AAV-Pf(s25-CSP) (n = 10 for both short-term and long-term studies) and Ad/AAV-Pf(s25-CSP) (n = 10 for short-term and n = 7 for long-term studies) after challenge with 2,500 PfCSP/Pb sporozoites. Sporozoite challenges were conducted at day 29 **(A)**, short-term) and day 102 **(B)**, long-term) after the last immunization. *P* values were calculated by log-rank (Mantel–Cox) tests versus the PBS group or each vaccine group. **(C, D)** IgG antibodies against PfCSP **(C)** and Pfs25 **(D)** in the protected mice in **(A)** were periodically monitored. *P* values were calculated by repeated measures two-way ANOVA with Sidak’s multiple comparisons test for the vaccine type. *p < 0.05; ****p < 0.0001.

Subsequently, the parasite burden was increased to 2,500 sporozoites during challenge, and the protective efficacy of m8Δ/AAV-Pf(s25-CSP) was compared with that of Ad/AAV-Pf(s25-CSP), an adenovirus-based vaccine that has previously demonstrated a durable antibody response (12). At day 29 after the last immunization, 10/10 mice (100%, *p* < 0.0001) were protected in the m8Δ/AAV-Pf(s25-CSP) group, while 7/10 mice (70%, *p* = 0.0031) were protected in the Ad/AAV-Pf(s25-CSP) group (**Figure 2A**). When challenged at day 102, 9/10 mice (90%, *p* = 0.0001) were protected in the m8Δ/AAV-Pf(s25-CSP) group, while 2/7 mice (29%, *p* = 0.1544) were protected in the Ad/AAV-Pf(s25-CSP) group (**Figure 2B**). After the challenge shown in **Figure 2A**, significantly higher IgG levels of both PfCSP and Pfs25 antibodies were durably induced in the m8Δ/AAV-Pf(s25-CSP)-vaccinated group than those in the Ad/AAV-Pf(s25-CSP)-vaccinated group for 210 days after the last immunization (**Figures 2C, D**). The induction of tissue-resident memory T cell immunity was also evaluated (14). The numbers of PfCSP-specific memory T cells, such as T_CM_ and T_EM_, in the spleen and liver of Ad/AAV-Pf(s25-CSP)-vaccinated mice were significantly higher than those of PBS control mice (**Supplementary Figures S7A, B**), while the total number of CD69^+^KLRG1^lo^ resident memory T cells (T_RM_) in the liver of m8Δ/AAV-Pf(s25-CSP)-vaccinated mice was significantly increased compared with the control group (**Supplementary Figures S7C, D**).

### Transmission blocking efficacy

**Figure 3:**
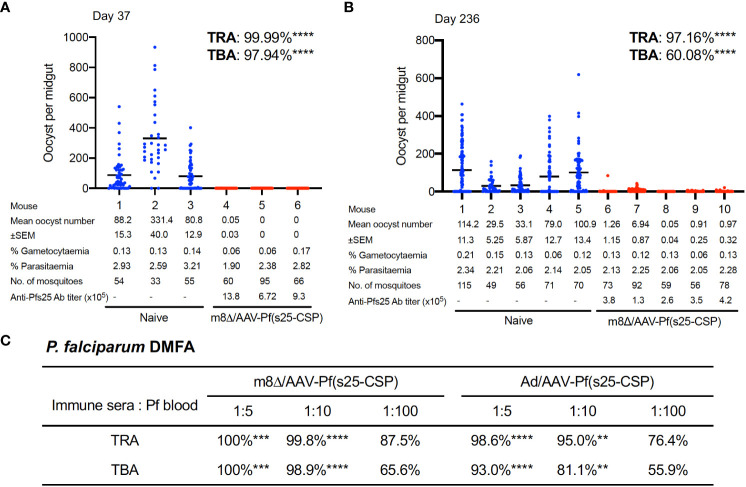
TB efficacy. **(A, B)** TB efficacy in mice immunized with m8Δ/AAV-Pf(s25-CSP) at day 37 **(A)**, (n = 3) and day 236 **(B)**, (wn = 5) after the last immunization. Each data point represents the oocyst number from a single blood-fed mosquito and horizontal lines indicate the mean number. **(C)** The sera from immunized mice at day 115 after the last immunization were tested for a direct membrane feeding assay. A summary of the transmission-reducing activity (TRA) and TB activity (TBA) for the indicated dilution ratio of the sera from m8Δ/AAV-Pf(s25-CSP) and Ad/AAV-Pf(s25-CSP) is shown. *P* values were calculated either using Mann–Whitney tests for TRA or Fisher’s exact probability tests for TBA versus the control groups. **p < 0.01; ***p < 0.0001; ****p < 0.0001.

TB efficacy was tested either 37 days (short-term) or 236 days (long-term) after the last immunization by a direct-feeding assay (19). In the short-term study, mosquitoes that fed on control mice displayed a mean intensity of 166.8 oocysts/midgut, while those that fed on the mice vaccinated with m8Δ/AAV-Pf(s25-CSP) had a mean intensity of 0.02 oocysts/midgut; thus, the vaccination achieved significant transmission-reducing activity (TRA) of 99.99% (*p* < 0.0001) (**Figure 3A**). The percentage of infected mosquitoes was reduced from 80.9% in the control group to 1.67% in the vaccinated group, achieving significant TB activity (TBA) of 97.9% (*p* < 0.0001) (**Figure 3A**). In the longer-term study, mosquitoes that fed on five control mice displayed a mean intensity of 98.02 oocysts/midgut, while the mean intensity of the vaccinated mice was 2.03 oocysts/midgut, achieving significant TRA of 97.2% (*p* < 0.0001) (**Figure 3B**). The percentage of infected mosquitoes was reduced from 67.1% of the control group to 26.8% of the vaccinated group, achieving significant TBA of 60.1% (*p* < 0.0001) (**Figure 3B**).

To assess the TB efficacy of m8Δ/AAV-Pf(s25-CSP) against field endemic *P. falciparum* at physiological parasite densities, a direct membrane feeding assay was carried out using *P. falciparum* gametocyte-positive blood from volunteers in Bobo Dioulasso, Burkina Faso. Sera from the m8Δ/AAV-Pf(s25-CSP) and Ad/AAV-Pf(s25-CSP) groups at day 115 following the last immunization were mixed with freshly-harvested gametocyte-positive blood and fed through an artificial membrane to female field-caught *Anopheles coluzzii*. In all three experiments, oocysts were observed in mosquitoes after the direct membrane feeding assay and following supplementation with sera from control mice that were immunized with m8Δ/AAV-empty vaccines (**Supplementary Figure S8**). By contrast, sera from m8Δ/AAV-Pf(s25-CSP) and Ad/AAV-Pf(s25-CSP) immunized mice provided significant TRA of 100% (*p* = 0.0002) and 98.6% (*p* < 0.0001) when diluted 1:5, 99.8% (*p* < 0.0001) and 95.0% (*p* = 0.0052) when diluted 1:10, and 87.5% (*p* = 0.7631) and 76.4% (*p* > 0.9999) when diluted 1:100 for the serum versus blood dilution ratio, respectively (**Figure 3C**). Similarly, sera of m8Δ/AAV-Pf(s25-CSP) and Ad/AAV-Pf(s25-CSP) provided significant TBA of 100% (*p* = 0.0003) and 93.0% (*p* < 0.0001) when diluted 1:5, 98.9% (*p* < 0.0001) and 81.1% (*p* = 0.0052) when diluted 1:10, and 65.6% (*p* = 0.1672) and 55.9% (*p* > 0.9999) when diluted 1:100, respectively (**Figure 3C**).

### Protective efficacy against multiple exposure, followed by mosquito biting challenge

**Figure 4:**
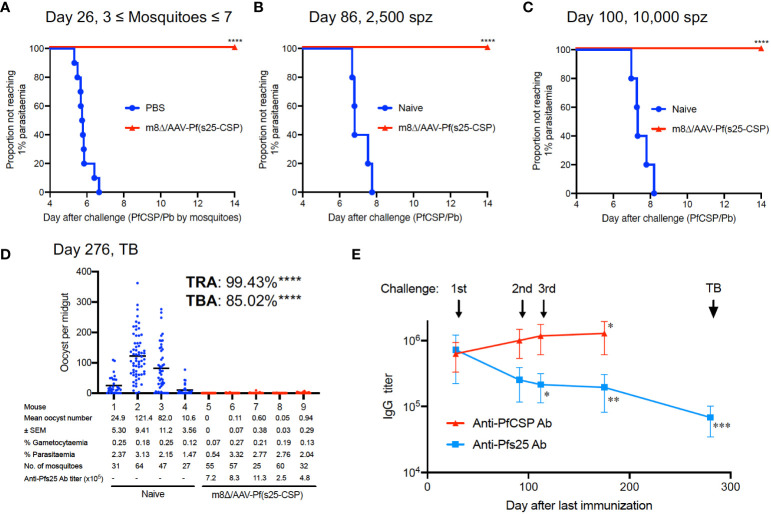
Multiple parasite exposure. **(A)** Protective efficacy in mice immunized with m8Δ/AAV-Pf(s25-CSP) (n = 10) after challenge by the bites of PfCSP/Pb sporozoite-infected mosquitoes at day 26 after the last immunization. **(B, C)** Protective efficacy in the protected mice in **(A)** against 2,500 **(B)**, day 86) and 10,000 PfCSP/Pb sporozoites **(C)**, day 100). **(D)** TB efficacy in the protected mice at day 276 (n = 5) versus naïve mice (n = 4). **(E)** IgG antibodies against PfCSP and Pfs25 at the indicated time points before each challenge and TB studies. *P* values (day 25 versus others) were calculated using ANOVA followed by Dunnett’s multiple comparisons test. *p < 0.05; **p < 0.01; ***p < 0.0001; ****p < 0.0001.

In malaria endemic areas, repeated exposure to *Plasmodium* is common, and therefore developing a vaccine with strong efficacy against repeated parasitic exposure is an obvious requirement. To verify this, m8Δ/AAV-Pf(s25-CSP)-vaccinated mice were first challenged by the bite of PfCSP/Pb-infected mosquitoes, and then repeatedly exposed to sporozoites by challenge *via* intravenous injection. In all, 10/10 mice (100%, *p* < 0.0001) were protected from the bites of infected mosquitoes (**Figure 4A**). The mean number of infected mosquitoes allowed to feed on individual mice was 5.2, a number previously shown to be 100% infectious (20) (**Supplementary Figure S9**). At day 86 and day 100 after the final immunization, the same mice were challenged intravenously with 2,500 sporozoites and 10,000 sporozoites, respectively. All vaccinated mice (100%, *p* = 0.0003) were protected at day 86 and day 100, while all control mice were infected (**Figures 4B, C**). Next, five of the multiple challenge protected mice were infected with blood-stage Pb-Pfs25DR3 parasites, and then examined for TB efficacy by direct feeding assays at day 276. Following mosquito feeding, the four control mice displayed a mean intensity of 59.7 oocysts/midgut, compared with 0.34 oocysts/midgut for the vaccinated mice, achieving significant TRA of 99.4% (*p* < 0.0001) (**Figure 4D**). The mean infected proportion was reduced from 81.0% of the control group to 12.1% of the vaccinated group, achieving significant TBA of 85.0% (*p* < 0.0001) (**Figure 4D**).

The IgG levels of PfCSP in the sera were significantly increased to 1,280,500 at day 175, compared with 631,750 at day 25 after the last immunization (*p* = 0.0279) (**Figure 4E**). Conversely, the IgG levels of Pfs25 in the sera were 721,540 units at day 25 after the last immunization, which gradually and significantly decreased to 194,507 units at day 175 (*p* = 0.0044) (**Figure 4E**). Frequent exposure to sporozoite challenge increased the IgG level of PfCSP, but decreased the IgG level of Pfs25. The IgG level of Pfs25 was finally measured at 68,195 units at day 272 (*p* < 0.0001 versus day 25), when the direct-feeding assay to assess TB efficacy was performed (**Figure 4E**). Despite the decrease in titer, strong efficacy (TRA of 99.4% and TBA of 85.0%) was still observed at this time point (**Figure 4D**).

## Discussion

The anti-malarial vaccine delivery system described here consists of two key technologies: a vaccine platform and a gene cassette. For the vaccine platform, a heterologous immunization regimen involving m8Δ-prime and AAV-boost induces long-lasting protective immune responses against *Plasmodium*. M8 is a licensed Japanese smallpox vaccine (21) used for smallpox eradication program led by the WHO. Vaccination of M8 to over 100,000 Japanese children did not lead to any severe post-vaccine complications (21). M8Δ is genetically a more-stable variant of m8 (6). The m8Δ-based vaccine vector induces antibody- and cell-mediated immune responses against foreign antigens approximately 500 times more efficiently than non-replicating modified vaccinia virus Ankara (MVA) (6). A homologous immunization regimen using m8Δ alone was examined as part of this work; however, it induced significantly lower IgG levels of anti-PfCSP antibody than other combinations, and protected only 20% of mice against sporozoite challenge (**Supplementary Figures S3A, E**). Thus, AAV was selected as a booster vaccine because of the potential of AAV vectors to induce durable immunity (13). AAV is a non-pathogenic virus that has been extensively used as a gene-therapy vector in clinical trials but has only recently emerged within the vaccination field (22, 23). A characteristic of AAV vaccination is the generation of a long-lasting antibody response due to long-term gene expression (12, 13), and the generation of a long-lasting immune response is currently a key challenge in the development of anti-malarial vaccines. Previously, a three-dose vaccination regimen with RTS,S/AS01 lost efficacy after 7 years (24) and R21/Matrix-M required four doses to maintain its efficacy for 1 year (25). Our data, and previous clinical studies using this platform, suggests that two doses of m8Δ/AAV vaccine would be sufficient to ensure high-level, long-term protective immune responses in humans. This remains to be tested in larger-scale clinical trials in the future.

The gene cassettes encoding PfCSP and Pfs25 linked *via* the hinge sequence, and the fusion protein, were introduced into an m8Δ/AAV vaccine, which enabled it to function as not only a pre-erythrocytic vaccine (PEV) but also a TB vaccine (TBV). In terms of a PEV, the method using transgenic rodent parasites such as PfCSP/Pb has been well established, but achieving complete protection in mice using these vaccines is challenging (26). In fact, it was necessary for sterile protection against the transgenic parasites to select a specific combination of the vaccines such as RTS,S and R21 along with potent adjuvants because other combinations resulted in only 0%–80% protection (27, 28). The regime tested here provided sterile protection of 90%–100% against challenge by intravenous injection of sporozoites, and against multiple challenges followed by bites from infectious mosquitoes. A fusion-type vaccine and a PfCSP stand-alone-type vaccine showed equally high efficacy (**Figure 1C**), with the antigenic structure of the fusion protein not detectably affecting the observed efficacy. Regarding Pfs25-based TBVs, a wide range of vaccine candidates/constructs have provided high functional titers and TB efficacy in mice but have resulted in limited success in human clinical trials (29–32). A viral-vectored TBV candidate, ChAd63/MVA Pfs25-IMX313, did not appear to induce strong antibody responses in humans, and this was associated with an inability to elicit CD4^+^ T cell responses, resulting in weak TB efficacy (33, 34). In our study, long-lasting antibody induction with a high level of anti-Pfs25 IgG by m8Δ/AAV was superior to that resulting from Ad/AAV vaccination (**Figure 2D**). We anticipate, based on previous studies, that the comparatively long-lasting gene expression by AAV (13) will allow for continuous exposure of antigen to MHC molecules in humans and induce strong IgG levels for TB activity throughout the transmission season in a malaria endemic region. There may be a concern that pre-existing immunity against AAV capsid may hamper the efficacy of the vaccine. In fact, a number of studies have demonstrated the efficacy of intramuscular injection of the vector even in the presence of neutralizing antibody against the capsid (35). In the case of intramuscular injection, safety profiles, including a lack of chromosomal integration, are being established through clinical and pre-clinical trials. Further studies, based around immunization in Controlled Human Malaria Infection, and decay in the functional titer, will be essential to examine these parameters in detail and to fully assess the overall public health impact of this delivery system.

An “ideal” malaria vaccine would have the ability to protect against repeated infections that are common in malaria endemic areas, and additionally reduce malaria transmission at the community level. These aims are clearly stated within the WHO Malaria Vaccine Roadmap, originally launched in 2006 and updated in 2013 (36). The multistage vaccine platform described in this study provides complete protection against three repeated sporozoite challenges over a period of 100 days following the final immunization (**Figures 4A–C**). Vaccination also conferred high TB activity for 276 days (**Figure 4D**). Specifically, the Malaria Vaccine Technology Roadmap focuses on developing safe and effective vaccines by 2030 that show at least 75% efficacy against clinical malaria and reduce transmission of the parasite by an unspecified amount (36). We have chosen PfCSP and Pfs25 as pre-erythrocytic and sexual-stage antigens, respectively, to develop a multistage vaccine effective both for protection and transmission blocking because these stages are parasite population bottlenecks in the human liver and the mosquito gut, as transmission occurs between human and insect hosts. Interactions between PEV and TBV could potentially enhance the efficacy of single vaccines, resulting in reduced parasite burden and even parasite elimination. This may prove a strong combination for a vaccine aiming to reduce malaria deaths in a natural community – especially if used over sequential transmission cycles (20). Based on the results of this animal study, we hypothesize that a multistage vaccine effective both for protection and transmission could have multiple advantages to overcome the potential risks of reducing vaccine effectiveness over time because of sequential gene mutation, polymorphism and subsequent pathogen escape.

In practical terms, a candidate anti-malarial vaccine must offer safety and efficacy during neonatal and early-life vaccination in resource-poor settings and would ideally be tailored for integration into current EPI vaccines. Host factors that may impair vaccine efficacy need to be taken into account. Malnutrition, helminth infection and maternal antibodies are critical host factors to be considered when developing a malaria vaccine (37, 38). Significant numbers of individuals living in tropical areas are co-infected with helminths, which adversely affect immune responses to a number of different existing vaccines (39). A vaccinia virus vaccine has been used extensively for immunization worldwide and is included in EPI vaccines used in infants in Africa without any interference from current EPI vaccines (40). The fourth-generation m8Δ is a highly-attenuated, safer variant (5, 6), and AAV induces long-lasting immune responses (12, 13). Therefore, m8Δ/AAV vaccination is expected to have protective efficacy that can last for a lifetime, particularly in infants.

Recently, more than 400 cases of monkeypox have been reported in 20 non-African nations, with most cases occurring in younger age groups (41). Historically, vaccination against smallpox was protective against monkeypox. However, the vaccine is no longer available after it was discontinued following global smallpox eradication (42). Because the parental strain m8 prevents lethal monkeypox (43), we speculate that our m8Δ-based malaria vaccine can prevent not only endemic malaria but also monkeypox outbreaks.

Here, we demonstrate the development of a next-generation multistage malaria vaccine effective against both pre-erythrocytic-stage and sexual-stage parasites based on the m8Δ and AAV viral platforms in a murine model. We report the successful implementation of the TBV element of our vaccine in a field setting and suggest the following four potential applications. First, it could effectively protect individuals against infection from mosquito bites by inducing sterilizing immunity. Second, it effectively reduces transmission of the native parasites through their natural vectors. If the same vaccine can induce similar responses in humans over successive transmission cycles, multiple models suggest it has the potential to result in elimination (20). Third, it is likely that our vaccine, based on the highly attenuated vaccinia strain m8Δ, could be safe and effective during neonatal and early-life vaccination in resource-poor settings and could theoretically be used in current EPI vaccines as a means of achievable and affordable malaria control. The use of a long-lasting, efficacious anti-malarial vaccine with multiple moieties against separate parts of the parasitic lifecycle to develop humoral and cellular based immunity, is an obviously attractive proposition for future clinical development. This strategy and unique delivery system could additionally be employed to induce immunogenicity against other antigens and may also be applied to other diseases of veterinary and clinical interest in the near future.

## Data availability statement

The raw data supporting the conclusions of this article will be made available by the authors, without undue reservation.

## Ethics statement

The studies involving human participants were reviewed and approved by The Centre Muraz Institutional Ethical Committee (Protocol 003-2009/CE-CM), Burkina Faso. The patients/participants provided their written informed consent to participate in this study. The animal study was reviewed and approved by The Animal Care and Ethical Review Committee of Kanazawa University (No. AP-214212) The University of Cambridge Ethical Review Committee (PPL PP8679814). Written informed consent was obtained from the individual(s) for the publication of any potentially identifiable images or data included in this article.

## Author contributions

Study concept and design: MI, AB and SY. Acquisition of data: MI, AB, TM, Y-IA, MN, NH, IY, DD, WG, AH, YY, AS, KY, HM, HS and SY. Analyses and interpretation of data: MI, AB, TM and SY. Drafting the manuscript: MI, AB and SY. Critical revision of the manuscript for important intellectual content: MI, AB, HM, HS and SY. Statistical analyses: MI, AB and TM. Technical or material support: MI, AB, HM, HS and SY. All authors contributed to the article and approved the submitted version.

## Funding

This work was partially supported by a Grant-in-Aid for Scientific Research (B) (JSPS KAKENHI grant number 19H03458) to SY, JSPS Bilateral Joint Research Projects (grant number JPJSBP120205704) to SY and a Grant-in-Aid for Scientific Research (C) (JSPS KAKENHI grant numbers 18K06655 and 21K06559) to MI. The research was also supported by the Global Health Innovative Technology Fund (grant number GHIT T2019-252) and the Japan Agency for Medical Research and Development (AMED) (grant number JP21nk0101539). AB was supported by the MRC [MR/N00227X/1], Sir Isaac Newton Trust, Alborada Fund, Wellcome Trust ISSF and University of Cambridge JRG Scheme, GHIT, Rosetrees Trust (G109130) and the Royal Society (RGS/R1/201293) (IEC/R3/19302). AH was supported by a MEXT fellowship (193298). The funding sources played no role in study design, the collection, analysis or interpretation of data, or publication.

## Acknowledgments

We thank R.E. Sinden for critical reading of the manuscript. We are grateful for the assistance of all members of the Laboratory of Vaccinology and Applied Immunology, Kanazawa University. The authors also thank A. Fujie, Y. Watanabe and E. Koyama for supporting this work and K. Fox from Edanz (https://jp.edanz.com/ac), for editing a draft of the manuscript.

## Conflict of interest

The authors have read the journal’s policy and declare the following conflicts of interest: Authors SY, HS, HM and MI are named inventors on filed patents related to viral-vectored malaria vaccines (2022-24221). HS is a named inventor on a filed patent related to LC16m8Δ (WO 2005/054451 A1). Neither of these products has been commercialized.

The remaining authors declare that the research was conducted in the absence of any commercial or financial relationships that could be construed as a potential conflict of interest.

## Publisher’s note

All claims expressed in this article are solely those of the authors and do not necessarily represent those of their affiliated organizations, or those of the publisher, the editors and the reviewers. Any product that may be evaluated in this article, or claim that may be made by its manufacturer, is not guaranteed or endorsed by the publisher.
