# Supplementary material for: Sterile protection and transmission blockade by a multistage anti-malarial vaccine in the pre-clinical study
Source: Front Immunol. 2022 Sep 29;13:1005476. doi: 10.3389/fimmu.2022.1005476 (PMC9558734; doi:10.3389/fimmu.2022.1005476)
Supplement: Supplementary file 1 [file DataSheet_1.pdf]

Supplementary Materials

Supplementary Figure S1

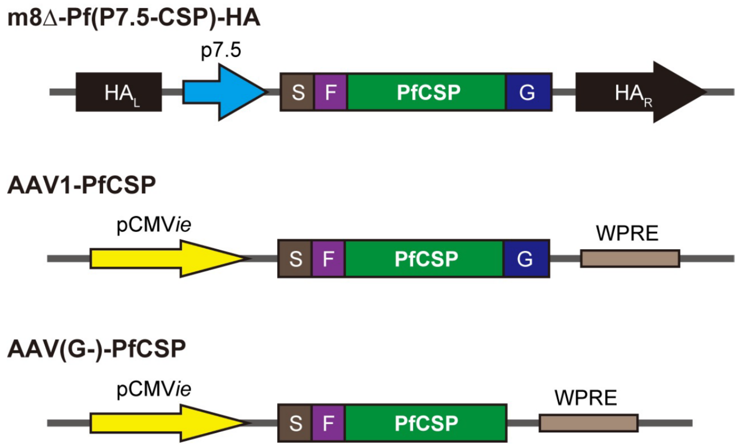

FIGURE S1. Schematic illustration showing the gene expression cassettes inserted into m8Δ-Pf(P7.5-CSP)-HA, AAV-PfCSP and AAV(G-)-PfCSP. In m8Δ-Pf(P7.5-CSP)-HA, expression of the *pfcsp* gene cassette was driven by the 7.5 promoter (p7.5) within the *hemagglutinin* (HA) gene. In AAVs, the immediate early CMV promoter (pCMV*ie*) was also present. S, signal sequence; F, FLAG epitope tag; G, the transmembrane region of VSV-G; WPRE, woodchuck hepatitis virus posttranscriptional regulatory element.

Supplementary Figure S2

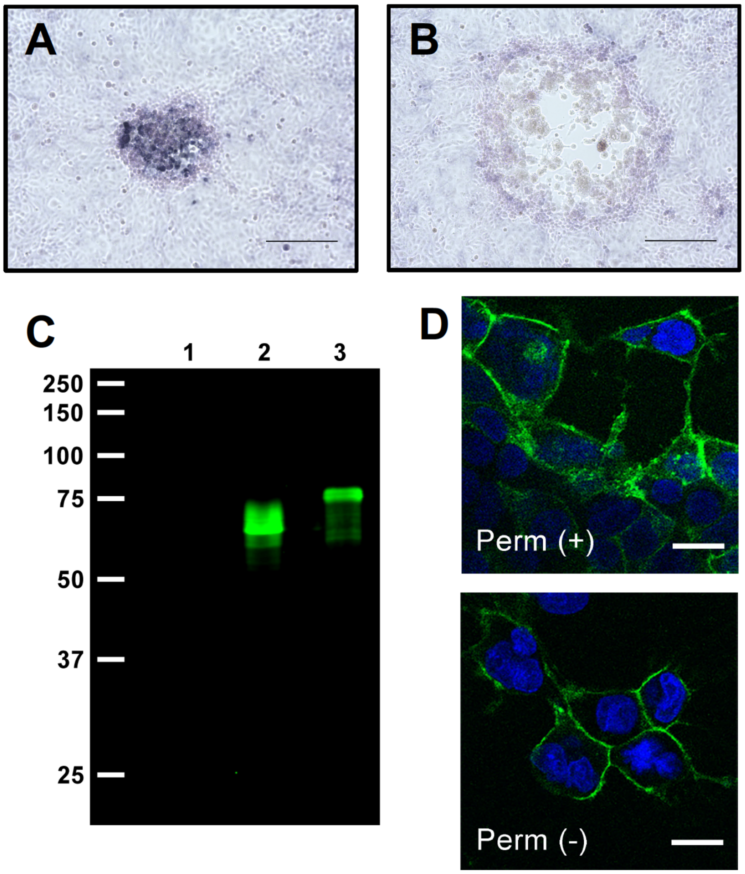

Figure S2. Construction of m8Δ-Pf(P7.5-CSP)-HA. (**A**, **B**) Representative plaques infected with either m8Δ-Pf(P7.5-CSP)-HA (A) or non-recombinant m8Δ (B) viruses and stained with anti-PfCSP mAb 2A10. Bars, 300 µm. (**C**) Analysis of PfCSP expression in HEK293T cells transduced with m8Δ-Pf(P7.5-CSP)-HA (MOI = 5). Cells were lysed and loaded onto a 10% SDS-PAGE gel and immunoblotted with 2A10 (Lane 1: Cells alone, Lane 2: Cells + m8Δ-Pf(P7.5-CSP)-HA). Thioredoxin-fused recombinant PfCSP (Lane 3: rPfCSP, an ELISA antigen) was loaded for the control. (**D**) Localization of PfCSP expression in mammalian cells after transduction with m8Δ-Pf(P7.5-CSP)-HA. After 24 h, the cells were fixed with either methanol [Perm (+)] or paraformaldehyde [Perm (-)], and then stained with Alexa Fluor-488-conjugated anti-PfCSP mAb (green). Cell nuclei were visualized with DAPI (blue). Original magnification, 400×. Scale bars = 20 µm.

Supplementary Figure S3

A

Day 24, 500 spz

Priming vaccine	Boosting vaccine	Protected mice / total mice (% Protective efficacy)
PBS	PBS	0/5 (0)
m8Δ-Pf(P7.5-CSP)-HA	m8Δ-Pf(P7.5-CSP)-HA	1/5 (20)
m8Δ-Pf(P7.5-CSP)-HA	AAV(G-)-PfCSP	2/5 (40)
AAV(G-)-PfCSP	m8Δ-Pf(P7.5-CSP)-HA	0/5 (0)

D

Day 24, 500 spz

Priming vaccine	Boosting vaccine	Protected mice / total mice (% Protective efficacy)
PBS	PBS	2/10 (0)
m8Δ-Pf(P7.5-CSP)-HA	AAV-PfCSP	8/10 (75)*

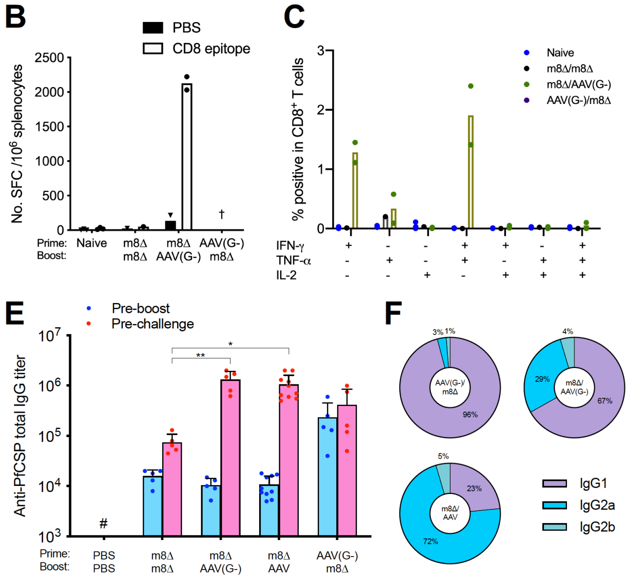

Figure S3. Preliminary studies of vaccine efficacy for m8Δ and AAVs. (**A**, **D**) Immunized mice were challenged with 500 PfCSP/Pb sporozoites at day 24 after the last immunization. *P* values were calculated using a Fisher's exact probability test in comparison with a PBS control group. (**B**) Spleens from protected mice in (A) were collected 2 weeks after challenge, and the PfCSP CD8 epitope peptide-specific IFN-γ-secreting T cells were assayed. (**C**) Percentages of cytokine-secreting $CD8^+$ T cells in the spleen by intracellular cytokine staining. Bars indicate the means. N = 1 to 3; †, no mice were protected in the group. (**E**) At 41 days after priming and 23 days after boosting, IgG levels of PfCSP in sera were measured by ELISA. Bars and error bars indicate the means and SD of the values, respectively. Pooled data from two independent experiments (A and D) are shown. *P* values were calculated by Kruskal–Wallis tests with Dunn's correction. (**F**) Proportion of subclass IgG in terms of titer after boosting immunization in (E). IgG1, IgG2a and IgG2b levels of PfCSP were measured, and their percentages of the whole in each vaccination were calculated according to the mean titers. M8Δ, m8Δ-Pf(P7.5-CSP)-HA; AAV(G-), AAV(G-)-PfCSP; AAV, AAV-PfCSP.

Supplementary Figure S4

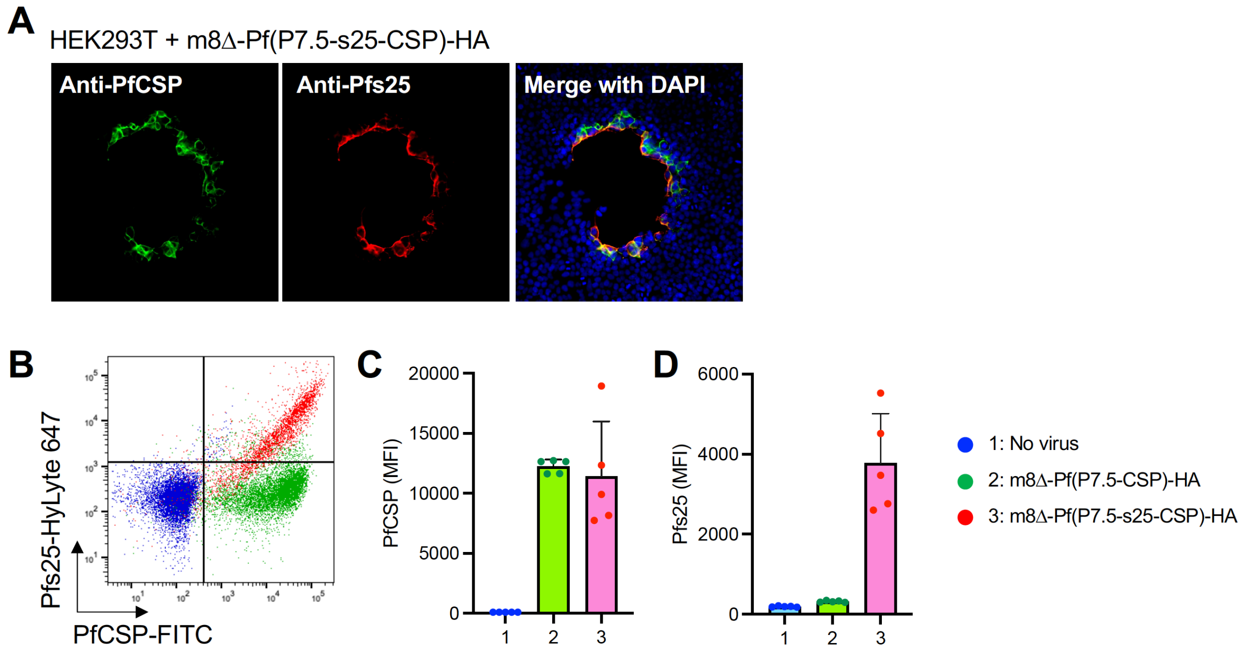

Figure S4. Protein expression by m8Δ-based malaria vaccines. HEK293T cells were transduced with m8Δ vaccines (MOI = 5) for 24 h. (**A**) Cells were transduced with m8Δ-Pf(P7.5-s25-CSP)-HA and stained with Alexa Fluor 488-conjugated anti-PfCSP mAb 2A10 and Alexa Fluor 594-conjugated anti-Pfs25 mAb 4B7 after fixation with methanol. (**B**–**D**) Cell-surface expression of PfCSP and/or Pfs25 was confirmed by flow cytometry. Cells were transduced with either m8Δ-Pf(P7.5-CSP)-HA or m8Δ-Pf(P7.5-s25-CSP)-HA and stained with FITC-conjugated 2A10 and HiLyte Fluor™ 647-conjugated 4B7. Merged data from representative wells (B) and the mean fluorescence intensity values and SD for PfCSP (C) and Pfs25 (D) are shown (n = 5).

Supplementary Figure S5

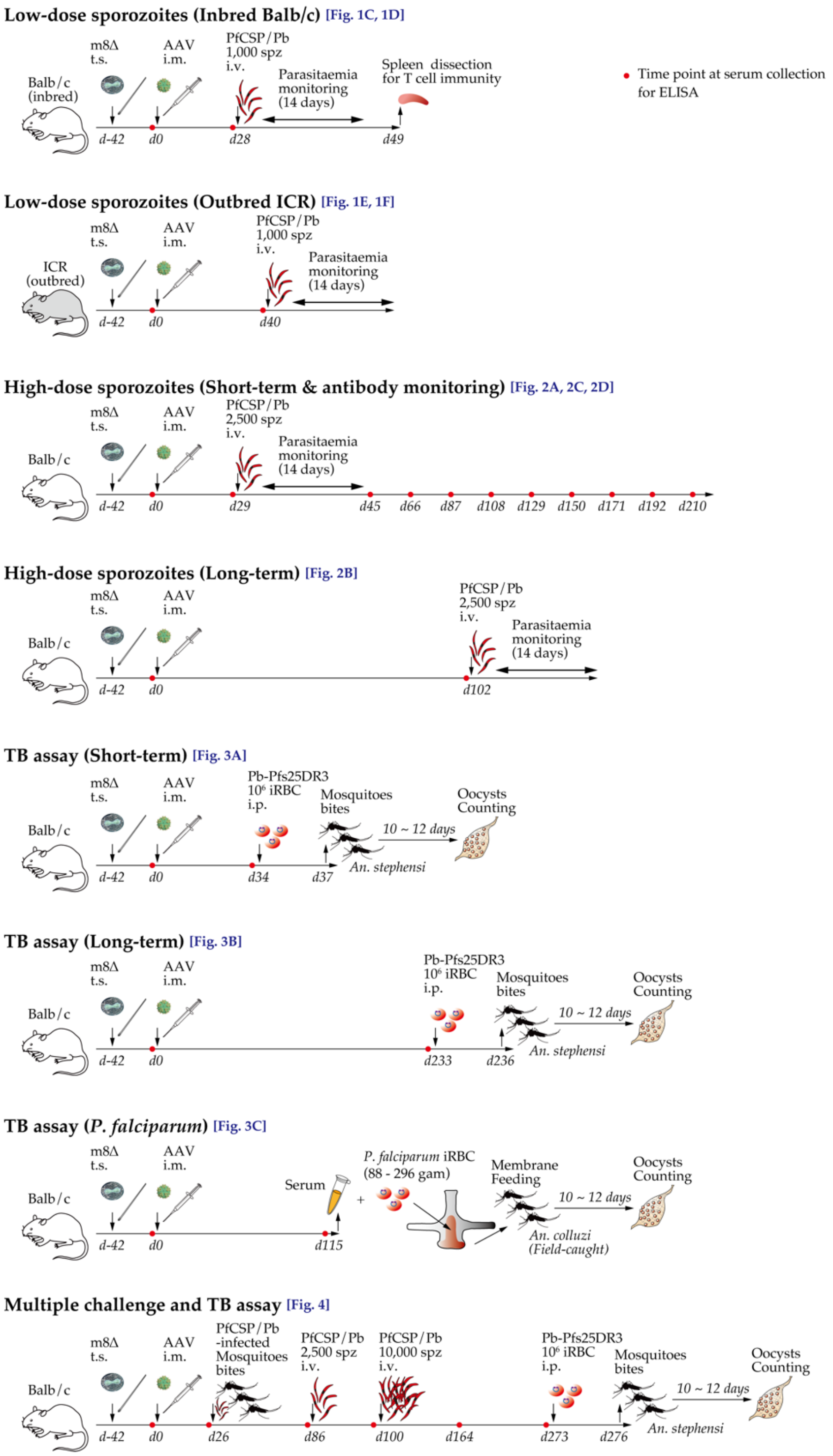

Figure S5. Experimental design of the sporozoite challenge experiments and TB assays. The time point for boosting immunization with AAV was set as day 0. Serum collection for ELISA was conducted one day before each intervention, i.e., immunization, sporozoite challenge and infection with infected red blood cells (iRBC) for TB assays, or periodically for long-term monitoring. T.s., tail scaring immunization; i.m., intramuscular immunization; i.v., intravenous injection; i.p., intraperitoneal injection.

Supplementary Figure S6

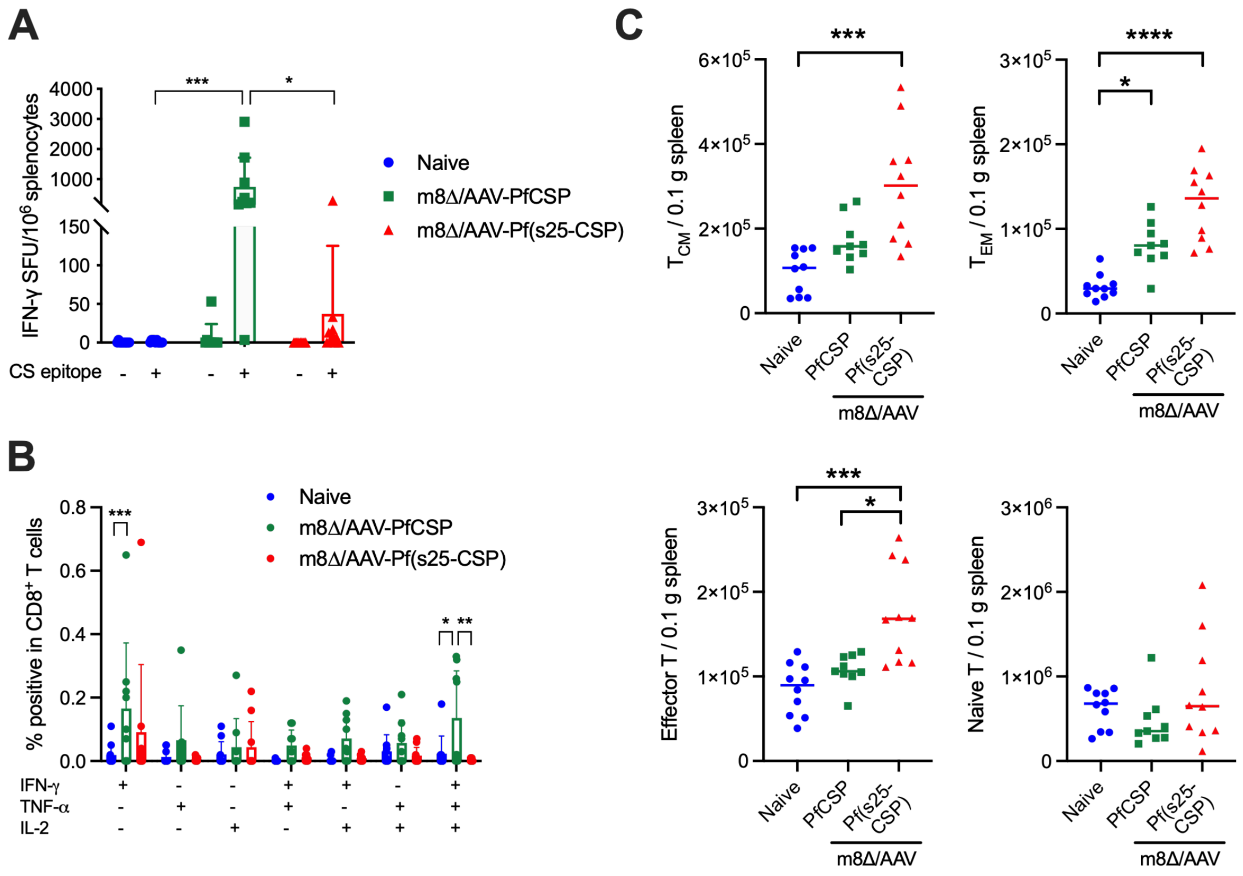

Figure S6. Cellular immune response in the mice that were protected from sporozoite challenge. Mice that were protected from sporozoite challenge in Fig. 1C were euthanized 5 weeks after challenge, and then the splenocytes were either stimulated with a CD8 epitope peptide of PfCSP for cytokine secretion or stained with markers for memory T cells (CD8, CD44 and CD62L). (**A**) Spot forming units (SFU) were determined for IFN-γ-secreting cells in an ELISpot assay. (**B**) Percentages of cytokine-secreting $CD8^+$ T cells in the spleen. (**C**) Numbers of central memory T cells (T_{CM}), effector memory T cells (T_{EM}), effector T cells and naïve T cells in the spleen. *P* values were calculated by Kruskal–Wallis tests with Dunn's correction for multiple comparisons.

Supplementary Figure S7

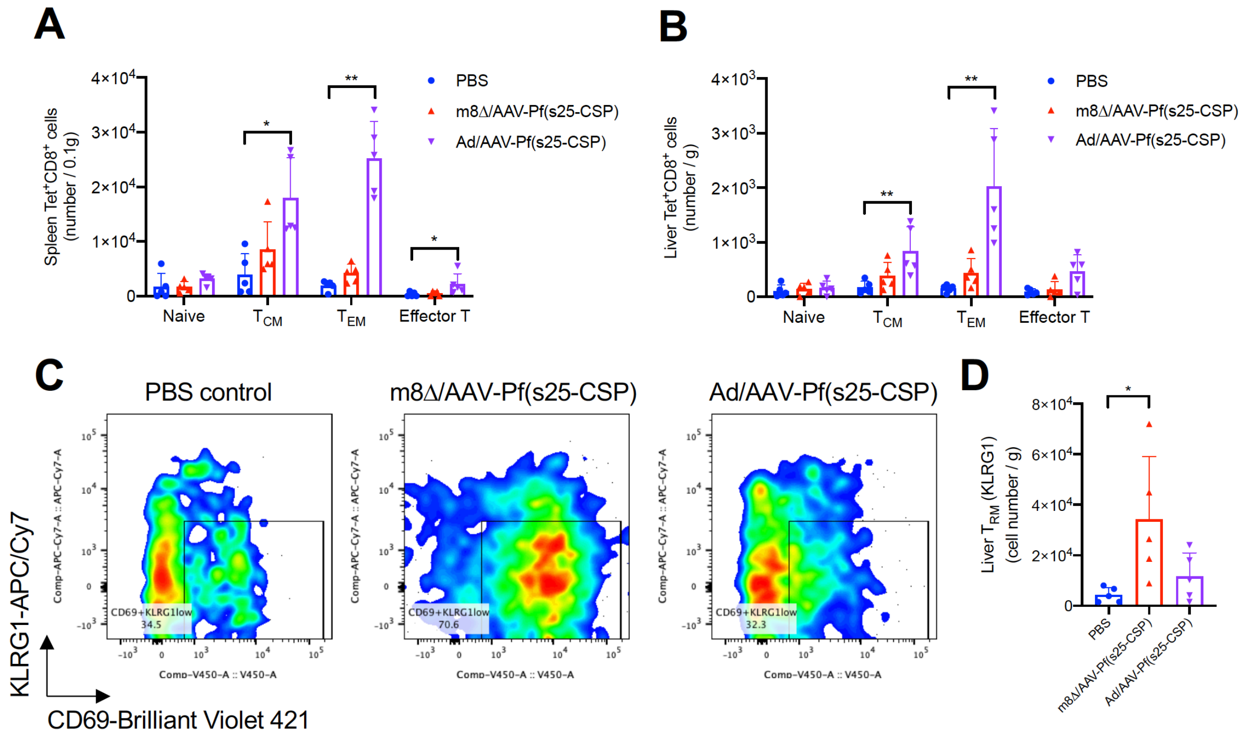

Figure S7. Memory T cells in the spleen and liver. (**A**, **B**) Vaccinated mice were euthanized 28 days after the last immunization, and then cells from either the spleen (A) or liver (B) were stained with markers for memory T cells (CD45, CD8, CD44 and CD62L) together with PfCSP epitope-bound MHC tetramer. (**C**) Representative plots of $CD69^{+}KLRG1^{lo}$ resident memory T cells (T_{RM}) in $CD44^{hi}CD62L^{-}$ effector memory T cells (T_{EM}) in the liver. (**D**) Total numbers of T_{RM} in the liver. *P* values were calculated by Kruskal–Wallis tests with Dunn's correction for multiple comparisons.

Supplementary Figure S8

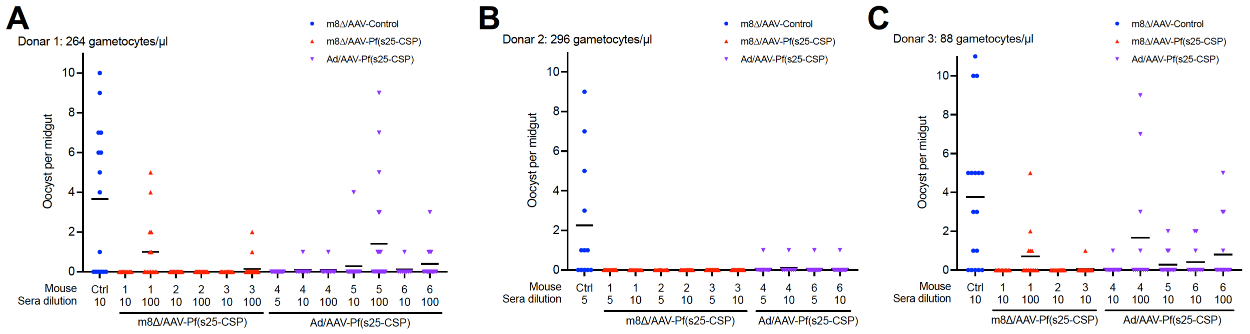

D

Priming vaccine	Boosting vaccine	Sera ID	Anti-Pfs25 titer
m8Δ-control	AAV-control	Ctrl	<100
m8Δ-Pf(P7.5-s25-CSP)-HA	AAV-Pf(s25-CSP)	#1	230,000
		#2	770,000
		#3	1,400,000
Ad-Pf(s25-CSP)	AAV-Pf(s25-CSP)	#4	87,000
		#5	95,000
		#6	128,000

Figure S8. Assessment of field-relevant TB efficacy by a direct membrane feeding assay. (**A**–**C**) The sera from immunized mice at day 115 after the last immunization were mixed with the gametocyte-positive blood at naturally-occurring parasite density from three independent donors, and the mixture was then fed on by field-caught *An. coluzzii*. At day 7 post-feeding, the mosquito midguts were dissected, and the parasite-positive proportion and oocyst intensity were determined. Each data point represents the oocyst number from a single blood-fed mosquito and horizontal lines indicate the mean number. (**D**) Anti-Pfs25 IgG levels of the sera are shown.

Supplementary Figure S9

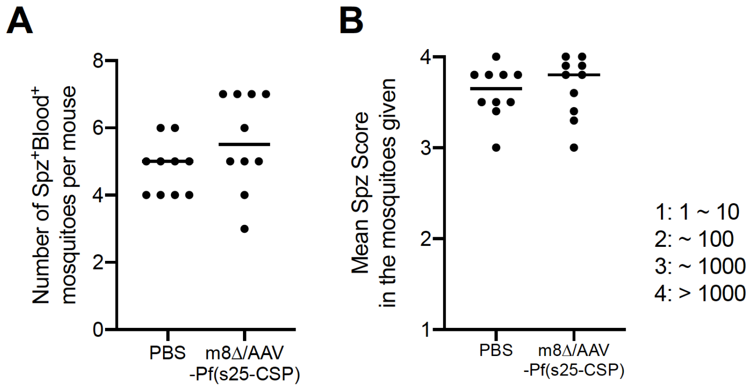

Figure S9. Infection status of mosquitoes used within challenge studies. Mosquitoes infected with PfCSP/Pb sporozoites were used in challenge infections of vaccinated mice. All the blood-fed mosquitoes were dissected for the salivary glands, and the infectivity of sporozoites was checked by microscopy. (**A**) The numbers of sporozoite-positive mosquitoes that fed on the blood from each mouse are shown. (**B**) Mean sporozoite scores in the mosquitoes that fed on one mouse are shown. A pair of salivary glands from individual mosquitoes was checked for the approximate number of sporozoites.
